# CT-derived Fractional Flow Reserve: How, When, and Where to use this Novel Cardiac Imaging Tool

**DOI:** 10.2174/011573403X300384240529124517

**Published:** 2024-06-04

**Authors:** Roozbeh Narimani Javid, Seyed Kianoosh Hosseini

**Affiliations:** 1 Student Research Committee, Hamadan University of Medical Sciences, Hamadan, Iran;; 2 Department of Cardiology, School of Medicine, Hamadan University of Medical Sciences, Hamadan, Iran

**Keywords:** Coronary artery disease, cardiac imaging techniques, fractional flow reserve, coronary angiography, computed tomography angiography, FFRCT

## Abstract

Fractional flow reserve computed tomography (FFRCT) is a novel imaging modality. It utilizes computational fluid dynamics analysis of coronary blood flow obtained from CCTA images to estimate the decrease in pressure across coronary stenosis during the maximum hyperemia.

The FFRCT can serve as a valuable tool in the assessment of coronary artery disease (CAD). This non-invasive option can be used as an alternative to the invasive fractional Flow Reserve (FFR) evaluation, which is presently considered the gold standard for evaluating the physiological significance of coronary stenoses. It can help in several clinical situations, including Assessment of Acute and stable chest pain, virtual planning for coronary stenting, and treatment decision-making.

Although FFRCT has demonstrated potential clinical applications as a non-invasive imaging technique, it is also crucial to acknowledge its limitations in clinical practice. As a result, it is imperative to meticulously evaluate the advantages and drawbacks of FFRCT individually and contemplate its application in combination with other diagnostic examinations and clinical data.

## 1 INTRODUCTION

Coronary artery disease (CAD) accounts for the primary cause of all deaths globally. CAD prevalence is continually rising, making it the leading cause of cardiovascular disease death worldwide [1]. The gold standard for diagnosing CAD is invasive coronary angiography (ICA), which is now enhanced by measuring fractional flow reserve (FFR) [2]. FFR is calculated by inducing maximal hyperemia in the coronary arteries and dividing the pressure distal to the obstruction by the pressure proximal to it (*i.e.*, typically pressure of the aorta) in order to identify any physiologically significant stenosis defined by an FFR of less than 0.80 [3]. Coronary revascularization guided by FFR is associated with lower mortality risks, myocardial infarction, and the need for emergent revascularization compared to ICA-guided revascularization or optimal medical therapy (OMT) alone [4]. However, utilizing FFR-guided ICA as an invasive approach can be expensive and result in procedure-related risks, such as bleeding and vasodilator side effects [5]. These limitations have prompted the investigation of less invasive alternatives for ICA, including coronary computed tomography angiography (CCTA).

CCTA reliably identifies and measures coronary plaque characteristics with a great negative predictive value (NPV) and an acceptable positive predictive value (PPV) relative to FFR-guided ICA [6, 7]. A non-invasive test should ideally offer anatomic and physiologic data to optimize specificity and sensitivity. This will ensure that patients with ischemia-causing lesions are treated appropriately while reducing the number of unneeded catheterizations. In this sense, CCTA is excellent at evaluating the anatomy of the coronary tree, but it lacks the ability to demonstrate the physiologic impact of a given anatomic stenosis. Moreover, the severity of coronary stenosis determined by CCTA is frequently overestimated, so only a portion of the severe stenoses detected in CCTA are accounted for myocardial ischemia [8].

To enrich CCTA findings, FFRCT was developed as a novel post-processing procedure on CCTA data, enabling it to predict the FFR and add valuable physiology information without additional invasive procedures.

This article comprehensively reviews FFRCT indications, clinical implications, and efficacy in various scenarios. We also briefly discuss the current limitations of using FFRCT.

## 2 HOW TO PERFORM FFRCT?

By utilizing computational fluid dynamics (CFD), Taylor *et al.* first introduced the concept of FFRCT, employing a computerized numerical simulation to accurately and non-invasively determine the value of FFR [9]. The incompressible Navier–Stokes equations were solved on computers as the foundation of CFD analysis. The investigators integrated simplified mathematical models of the heart, systemic circulation, and coronary microcirculation with personalized models of the aortic root and epicardial coronary arteries. These personalized models were created using obtained data from CCTA. Consequently, a geometric multi-scale coronary artery simulation was designed to achieve non-invasive computation of FFR. However, since the coronary artery network is a three-dimensional (3D) structure, a number of early FFRCT calculation systems, including HeartFlow, were done by solving the complete 3D version of the equations, which could take hours of computing time.

Afterward, some attempts were made to calculate the FFRCT using one-dimensional (1D) Navier–Stokes equations. The Siemens cFFR [10] and Toshiba CTFFR [11] systems utilized this less time-consuming method, called a reduced-order model. 3D and 1D FFRCT calculation comparisons have demonstrated similar outcomes [12], even for a complicated aortic flow [13]. Machine learning (ML) approaches have also been investigated to accelerate the process further. ML-FFRCT utilizes artificial intelligence algorithms to integrate the intricate, nonlinear connection between the data derived from the geometry of the coronary artery tree and to calculate the functional severity of the underlying coronary lesion. Most of these deep learning methods use a multi-layer neural network architecture that is trained offline to acquire knowledge about the complex relationship between the structure of the coronary tree and its corresponding hemodynamic properties. Using a computational fluid dynamics simulation, the ML model is then trained using a collection of artificially created coronary anatomies to identify the variations in anatomy and their corresponding hemodynamic circumstances. The ML-predicted FFRCT is generated in approximately 80 times less computational time than the reduced-order (1D) CFD-based FFR, with similar FFRCT results and diagnostic accuracies [14].

Consequently, due to the need for rapid analysis in clinical settings, the use of reduced order models and ML may become more prevalent than 3D computation in the future. In addition, a careful evaluation is necessary to determine the proper acceleration strategy according to the available inputs, their quality, and the desired outputs.

## 3 HOW MUCH IS THE DIAGNOSTIC ACCURACY?

Multiple trials have investigated the sensitivity, specificity, and accuracy of FFRCT with a threshold of 0.80 in detecting ischemia-causing CADs. The Discover FLOW trial was a prospective study that involved 103 patients with suspected and diagnosed CAD. The patients underwent CCTA and FFRCT, and the results were subsequently compared to the ICA-derived FFR. The FFRCT exhibited a sensitivity of 87.9%, specificity of 82.2%, and accuracy of 84.3% in comparison to the CCTA, which demonstrated a sensitivity of 91.4%, specificity of 39.6%, and accuracy of 58.5% [15]. DeFACTO was the next prospective study of 252 CAD patients conducted in the same manner as the preceding study, with almost comparable results [16].

**Fig. (1):**
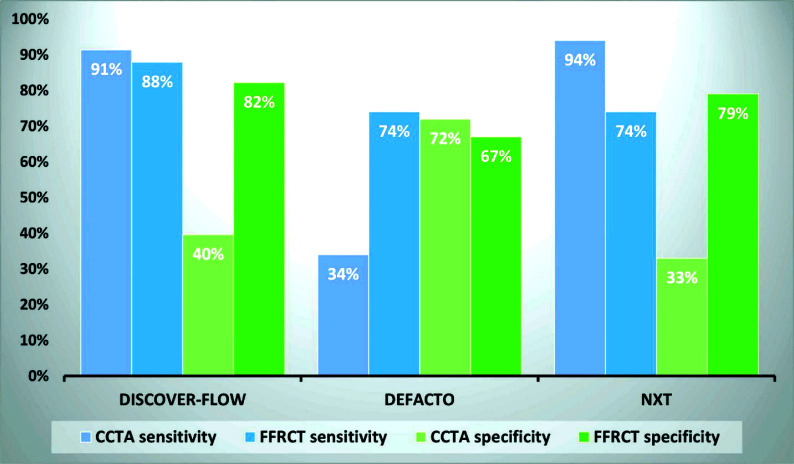
Comparison of diagnostic performance of coronary computed tomography angiography (CCTA) and fractional flow reserve computed tomography (FFRCT) in Three major primary trials including DISCOVER-FLOW, DeFACTO, and NXT trial.

Finally, the NXT trial, a similar prospective multicenter study involving 254 patients, demonstrated that the sensitivity, specificity, and accuracy of FFRCT were 88%, 79%, and 81%, respectively, compared to 94%, 33%, and 55%, for CCTA. Thus, the DISCOVER FLOW, DeFACTO, and NXT trials demonstrated an increase in both the specificity and accuracy of FFRCT compared to CCTA [15-17]. These three studies eventually led to FDA approval (DEN130045) of FFRCT generated by HeartFlow (Fig. **1**).

The machine learning approach in a study also demonstrated an acceptable sensitivity and specificity of 87% and 77%, respectively, using invasive FFR as a reference [18]. A summary of some important studies is presented in Table **1**.

### 3.1 Effect of Assessment Point on Diagnostic Accuracy

It has been shown that FFR decreases gradually from the ostium to the distal artery, even in normal blood vessels [19]. FFRCT value also demonstrates a similar progressive reduction. However, it is almost constant until 10.5 mm post-stenosis [20].

A study found that 44% of patients with a positive FFRCT for stenosis based on the lowest FFRCT in the artery (*i.e.*, in the distal artery) would have been redefined as negative when the FFRCT was assessed just distal to the stenosis. In patients who ultimately underwent ICA, those with a positive FFRCT distal to the stenosis were substantially more likely to receive revascularization than those with a positive FFRCT only in the distal artery (53% *vs*. 44%). So, according to this study, FFRCT and invasive FFR results have a stronger correlation when FFRCT is measured just distal to the stenosis [21]. By adopting this approach, the overestimation of the hemodynamic impact of stenosis would also be minimized [19, 20].

Consequently, 1-2 centimeters distal to the stenosis is the optimal location for calculating FFRCT rather than the distal artery [22]. In this way, the reduced accuracy reported by FFRCT in the reassess study, which measured FFRCT at the distal artery instead of just distal to the stenotic lesion, may be partially explained [23].

### 3.2 Delta FFRCT

Delta FFRCT (ΔFFRCT) is defined as the difference between FFRCT distal and proximal to a stenotic lesion. It can help assess the hemodynamic significance of stenosis more accurately by estimating the lesion-specific pressure loss [24, 25] and a ΔFFRCT of 0.12 or higher, indicating a significant impact on coronary hemodynamics [26]. Additionally, coronary plaques above the measurement point can affect the calculation of FFRCT, as they could lead to a drop in FFRCT before the stenosis and hinder the identification of its physiological significance. However, ΔFFRCT is not impacted by coronary plaques upstream of the stenosis [27]. Thus, utilizing ΔFFRCT is more effective in identifying patients needing early revascularization than the standard diagnostic approach using CCTA and FFRCT. This approach is particularly advantageous for patients with grey-zone FFRCT values between 0.76 and 0.80 [28].

## 4 WHEN TO USE FFRCT?

### 4.1 Stable Chest Pain in Intermediate-Risk Patients

Over the last two decades, recommendations for evaluating and treating CAD have undergone substantial modifications. Prior to the revision of the guidelines in November 2016, the UK National Institute for Health and Care Excellence (NICE) had recommended obtaining a thorough history of symptoms when investigating stable chest pain. Additionally, to select the best non-invasive diagnostic test, an assessment of the pre-test probability using the modified Diamond-Forrester model was necessary [29, 30]. However, the modified 2016 NICE guideline was remarkable for eliminating the pre-test probability model and recommending CCTA as the primary study for all patients with typical or atypical angina and also asymptomatic patients with suspicious ischemia-related ECG abnormalities [31]. On the other hand, while CCTA is helpful, it tends to overestimate the significance of CAD, and there is only a modest correlation between stenoses and subsequent myocardial ischemia [7]. Therefore, further stress testing is recommended for patients with equivocal symptoms and suspected obstructive CAD on CCTA [32].

Meanwhile, the positive short-term clinical outcome in patients with equivocal CCTA and FFRCT >0.80 in the PLATFORM trial [33] demonstrated that such patients can safely be discharged without requiring subsequent ICA or additional testing. We believe this result was due to the considerable negative predictive value of FFRCT for detecting ischemia [15, 17].

Thus, FFRCT is now considered a valuable diagnostic tool for stable angina in various guidelines.

According to the 2022 Japanese Circulation Society (JCS) guideline, FFRCT can be used alongside other non-invasive imaging techniques as an alternative approach for assessing stable coronary artery disease. As a result, when the CCTA indicates the existence of obstructive CAD, excluding the LMCA (Left Main Coronary Artery) or LMCA-equivalent disease, it is advisable to do further functional testing, such as stress imaging test and FFRCT, to further evaluate the risk before proceeding to invasive coronary angiography. The NICE guideline also recommends using HeartFlow FFRCT as a non-invasive method for assessing angina in patients with stable, recent-onset chest pain suspected of being of cardiac origin. It also states that HeartFlow FFRCT provides the clinician with additional functional information to identify the coronary stenoses causing myocardial ischemia. Additionally, the American Heart Association (AHA) Guideline for the Evaluation and Diagnosis of Chest Pain (2021) recommends that FFRCT may serve as a valuable diagnostic tool for identifying vessel-specific ischemia in intermediate-high risk patients presenting with stable chest pain and a previously diagnosed coronary stenosis ranging from 40% to 90% in a proximal or middle coronary segment on CCTA [34].

However, Since FFR contains a “grey zone” value for ischemia, making a definitive diagnosis in certain cases might be challenging. A lower accuracy has been shown for FFRCT when values are around the threshold of 0.80, while FFRCT values of more than 0.90 or less than 0.60 were found to provide almost perfect confidence [35]. A subsequent investigation of lesions incorrectly diagnosed by FFRCT also demonstrated that the false negative rate of FFRCT was about 5.9% in lesions with an FFR of less than 0.76, while the rate was as high as 20% for grey zone lesions. Calcified plaque also significantly increased the chance of FFRCT false-negative outcomes for lesions in the grey zone [36]. Regarding these limitations, the results from Scot-heart [37] and Ischemia [38] suggested that FFRCT analysis should only be applied to patients with inadequate symptomatic response to optimal medical treatment rather than using it based on the measure of atheroma discovered on CCTA.

**Fig. (2):**
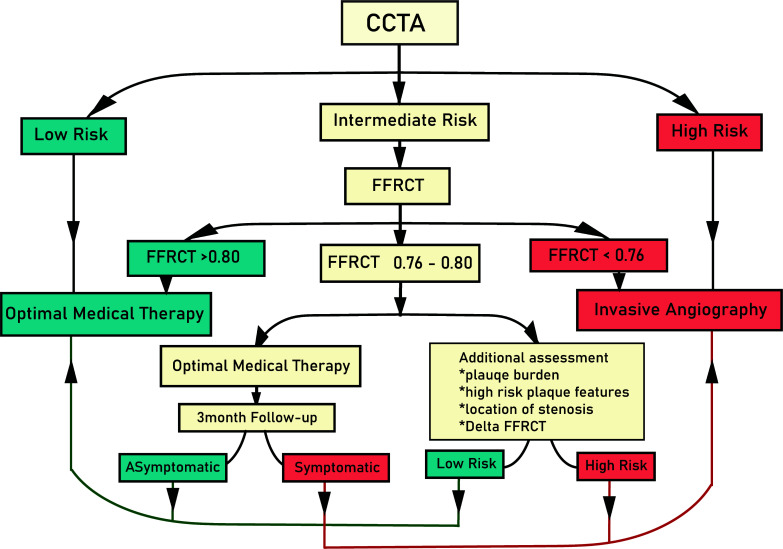
The algorithm for approaching a patient with stable chest pain. After performing CCTA as the primary imaging, risk stratification is done based on its findings.

The first flowchart for utilizing FFRCT in patients with stable chest pain and intermediate risk was introduced by Nørgaard *et al.* [39]. Later, it underwent some changes due to a new study [40] and was modified by Rajiah *et al.* [41]. The modified flowchart advises that decisions about a referral to ICA for patients with FFRCT between 0.76 and 0.80 should be made based on any available data like plaque burden, stenosis location, Delta FFRCT, and the patient's response to an experimental three-month period of Optimal medical therapy (OMT). At the same time, it recommends OMT or ICA for patients with FFRCT > 0.80 or ≤ 0.75, respectively. A number of factors supports the importance of this strategy. First off, those suffering from stable chest pain have a generally good prognosis in current medical practice [33, 42], and the average annual risk of cardiac mortality or myocardial infarction following deferral of coronary revascularization in patients with intermediate-range lesions and FFRCT ≥0.75 is extremely low (1%) [43]. On the other hand, when the FFRCT is less than 0.75, the possibility of the need for revascularization will increase significantly [39]. However, further investigations are necessary to define the safety of this FFRCT-guided diagnostic strategy in symptomatic patients suffering from a moderate coronary lesion (Fig. **2**).

### 4.2 Acute Coronary Syndrome

According to prior studies, employing CCTA in the assessment of acute chest pain in the Emergency Department (ED) may result in quicker diagnosis and reduced cost of treatment when compared to SPECT. There are no differences in MACE between these two strategies in 6-month follow-up [44, 45]. However, several studies also showed an increased need for secondary evaluations, including additional functional testing and ICA among CCTA patients [42, 45, 46]. FFRCT can assist in reducing unnecessary ICA and further tests, even in acute settings. A recent study [47] has demonstrated that patients presented to the ED with acute chest pain who underwent CCTA with an FFRCT of >0.8 are highly safe against experiencing any MACE at 30 days. However, the patients with STEMI, NSTEMI, and hemodynamic instability were excluded from this study. Some patients with an FFRCT of >80 were also admitted to the hospital and underwent further investigations, including ICA. However, the results returned negative for any significant obstructive disease.

While several studies have shown the value of FFRCT in patients with stable chest pain, Investigations on implementing this strategy in acute settings are still in their early stages. Thus, Additional studies are necessary to determine if FFRCT can be used as a risk stratifier for patients with acute chest pain. This will help identify which patients require further invasive care and which can be safely discharged.

On the other hand, multivessel CAD affects about 40% of ACS patients [48]. Clinical investigations have shown that those with ACS who still have one or more functionally significant non-culprit lesions (NCLs) following primary PCI are at an increased risk for secondary cardiovascular events [49, 50]. As a result, total revascularization is preferable to a culprit-only revascularization approach in this group of patients [51, 52]. However, the proper approach and time for determining an NCL's need for revascularization are still up for debate [53-55]. For the functional evaluation of NCLs in ACS patients, invasive FFR and non-invasive dobutamine stress echocardiography (DSE) are commonly accepted and advised approaches [3, 49, 50].

Gaur *et al.* performed the first major study to assess the diagnostic accuracy of FFRCT in diagnosing lesion-specific ischemia in non-culprit arteries of patients who recently had STEMI [56]. Researchers assessed 124 non-culprit vessels obtained from a cohort of 60 patients. Each patient's total coronary artery lumen volume was divided by the left ventricular myocardial mass to determine the vessel volume relative to myocardial mass (volume-to-mass ratio), which was used to correct the vessel lumen volume for variations in the size of the supplied myocardium. The FFRCT demonstrated an accuracy of 72%, a sensitivity of 83%, and a specificity of 66%. The present study demonstrated that the diagnostic accuracy of FFRCT for detecting ischemia in STEMI patients with multivessel disease is modest since it is affected by the volume-to-mass ratio and the fact that STEMI patients have reduced vessel volume compared to patients with stable angina.

A recent prospective investigation [57] has also been carried out to determine the efficacy of on-site FFRCT in assessing concurrent NCLs in ACS. According to study results, on-site FFRCT has a generally acceptable diagnostic performance in predicting the hemodynamic significance of NCLs in ACS patients. However, its sensitivity and specificity were relatively lower than invasive FFR (51% and 89%, respectively) but much higher than conventional CCTA. These findings suggest that, at this level of development, on-site FFRCT may not be accurate enough to serve as a surrogate of the invasive functional assessment in the case of NCLs, but it still can play its role in stratifying the patients' risk at ED.

### 4.3 Detecting Post-stenting Restenosis

Despite the positive long-term results of FFR-guided PCI, the FAME study found that MACEs occurred in 13.2% of patients at one year and in 20% at two years after PCI in the FFR group [3, 58]. According to intravascular ultrasonography, in-stent restenosis (ISR) occurs in 20-40% of patients treated with bare-metal stents (BMS) [59, 60] and in 5-10% of patients treated with drug-eluting stents (DES) [61]. Because of this, evaluating patients with earlier PCI requires a reliable and accurate method to assess a possible ISR. Invasive FFR is rarely used for post-procedural assessments. This is partly because of the risks related to this procedure's invasive and costly nature [62].

Although CCTA is an effective diagnostic tool for CAD, it should be avoided in patients with intracoronary stents due to the artifacts caused by the stent's metallic design [63]. The role of FFRCT for its potential utility in assessing patients who already have an intracoronary stent has also been investigated. A novel study [64] on patients from the CHINA FFRCT investigation [65] with prior coronary stent placement and a following invasive FFR at least three months later demonstrated a good correlation between FFRCT and invasive FFR. In post-PCI patients, FFRCT was shown to have an accuracy of 85.7% in detecting hemodynamic ISR while using invasive FFR as the reference standard. According to the high association between FFRCT and invasive FFR in patients following stent implantation, Follow-up assessment of these patients with FFRCT is now a feasible option. However, this study's limited sample size necessitates additional studies with larger sample sizes to reevaluate the diagnostic efficacy of FFRCT in detecting hemodynamic ISR in daily clinical practice.

### 4.4 Preoperative Assessment of CABG Candidates

Invasive coronary angiography has historically served as the primary surgical guidance in patients with complicated multivessel CAD undergoing coronary artery bypass graft (CABG) [66]. Several studies, however, revealed that ICA might not be accurate in detecting stenosis that causes ischemia [67, 68]. Competitive flow is the leading cause of graft failure and contributes to the loss of more than 75% of the internal thoracic artery (ITA) grafts due to bypassing non-functional stenoses [69]. Additionally, up to 25% of grafts failed to show regional myocardial perfusion improvement after CABG [70]. Therefore, conducting a functional evaluation before planning CABG for a patient is crucial.

FFR-guided CABG is linked to fewer complications, greater graft patency rate, and other clinical advantages compared to an angiography-guided technique [71, 72]. The downsides of FFR, however, include the fact that it is more expensive, exposes patients to radiation, and involves invasive procedures [73]. On the other hand, the ability of FFRCT as a non-invasive tool to identify CABG candidates and determine operation approach has been supported by current guidelines due to a number of studies [74-77]. According to one study, cardiac surgeons felt confident making a surgical choice only based on FFRCT results in 84% of clinical scenarios [75].The earliest case of successful CABG with FFRCT as the only guiding tool has been reported recently [74]. In this case, multiple FFRCT calculations were also done post-operatively to evaluate the functional improvement. Also, the ongoing fasttrack CABG trial [78] is going to assess the reliability and feasibility of planning CABG mainly based on CCTA and FFRCT, regardless of ICA results.

Preoperative FFRCT can also help determine anastomosis patency or obstruction. It has been shown that FFRCT values ≤0.80 were connected to a 90% decrease in the relative risk of anastomosis obstruction in an average follow-up of 15.3 months [79]. This may be explained by the fact that the success of a coronary artery bypass depends on the graft flow exceeding native coronary flow. With less severe ischemia, flow competition between native arteries and graft conduits may intensify, leading to a drop in flow demand for graft, which is the primary cause of graft failure and may accelerate the atherosclerosis process in native coronary arteries [80-82]. As a result, with the inclusion of FFRCT in the preoperative assessment for CABG, the anastomosis obstruction rate will be dramatically decreased without adding further risk of invasive procedures.

### 4.5 Virtual Coronary Stenting

As soon as the stent is implanted, FFR may be used to evaluate the accomplished functional revascularization. The likelihood of negative outcomes after PCI varies based on the degree of functional revascularization achieved [83, 84], and the prognosis of patients with higher FFR levels after PCI is more favorable than patients with lower post-PCI FFR [85, 86]. Nevertheless, it is remarkable that about a third of patients still have inadequate FFR values following an angiographically effective PCI [87, 88]. As a result, methods that can help improve functional revascularization may have the ability to improve PCI results.

The value of CCTA in guiding and planning procedures is becoming more widely recognized. The FFRCT Planner is an advanced CCTA-based program that enables physicians to simulate the stenting of coronary stenoses. Consequently, the post-PCI FFR can be predicted virtually [89]. It has been demonstrated that in the cases with invasive FFR of ≥0.80 and ≥0.90, the FFRCT Planner can correctly estimate post-PCI FFR with an accuracy of 83% and 71%, respectively. There is also a significant agreement between the luminal dimensions obtained invasively using optical coherence tomography (OCT) post-PCI and those obtained following stenosis remodeling on the FFRCT model [90].

**Fig. (3):**
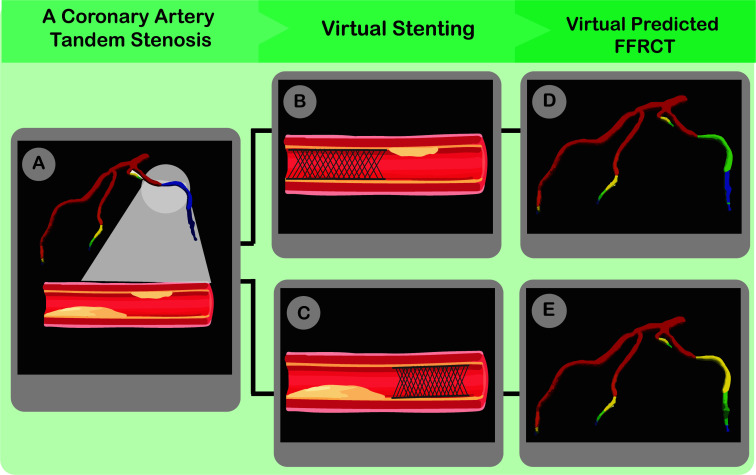
FFRCT can be used as a virtual planner. In a given tandem stenosis (**A**), the cardiologist can virtually stent any part of the stenosis (**B**, **C**) and see the predicted FFRCT (**D**, **E**) immediately. As a result, the best length and place of stenting to obtain maximum FFRCT will be selected.

This program makes it possible to arrange PCI preoperatively in a previously impossible way. Additionally, regardless of the primary CAD pattern (*i.e.*, focal or diffuse), the level of calcification, or the resolution of the CT images, the accuracy of the FFRCT Planner remains untouched [90]. As a result, the FFRCT Planner may aid Cardiologists in selecting patients more effectively for invasive procedures, avoiding pointless PCIs, and estimating the possible benefits of interventions. This technology can play additional important roles in the Cath lab. For instance, in patients with tandem stenosis, the FFRCT Planner can aid in determining the accomplished incremental benefits after stenting each lesion. So, in cases with diffuse disease, it helps to get the greatest possible benefit out of the intervention while limiting complications and stent length [91]. However, like other FFRCT applications, the FFRCT planer's efficacy must be verified by additional investigations (Fig. **3**).

### 4.6 As a Decision-Making Tool

The SYNTAX III REVOLUTION showed that medical decisions based on CCTA are highly consistent with those obtained from standard coronary angiography in patients with left main or three-vessel disease (3VD) [92]. Utilizing FFRCT in addition to CCTA can also modify the selection of treatment options.

The role of FFRCT in making treatment decisions and selecting the procedure type and strategy has been investigated among cardiac teams of patients with left main or 3VD. It was shown that the cardiac teams' treatment decisions between PCI and CABG were revised in 7% of situations, and the selected arteries for revascularization were altered in 12% of cases when the functional assessment with FFRCT was included in comparison to a CCTA assessment alone. In addition, adding FFRCT data to standard angiography also altered the recommendation for treatment in 6.6% of the patients and revised planning in 18.3%. FFRCT also decreased the percentage of patients who were considered to have hemodynamically significant 3VD by CCTA from 92.3% to 78.8% [93].

Therefore, by adding the functional aspect of coronary artery lesions, FFRCT can improve the cardiac teams' decisions and planning process. In addition, the quite low radiation exposure dosage feasible with the newest generation of scanners [94] makes FFRCT a promising non-invasive tool for assessing patients with multivessel disease prior to a revascularization procedure, thanks to its remarkable accuracy in this group of patients.

## 5 WHAT ARE THE OVERALL ADVANTAGES OF USING FFRCT?

### 5.1 A Gatekeeper for the Cath Lab

The primary advantage of FFRCT, as established in several prior studies, has been the reduction in unnecessary ICA [95]. In the platform trial, the researchers showed that using an FFRCT-guided technique resulted in a lower referral rate for cardiac catheterization, and the patients who finally underwent ICA were more likely to have obstructive CAD [33, 95]. The favorable outcome of this pilot trial encouraged additional clinical investigations.

The advance study [96] was designed to investigate the practical applicability of FFRCT. In this trial, FFRCT reclassified patients and altered treatment strategy in 66.9% of cases. The study also showed that in Patients with FFRCT≤ 0.80, after performing ICA, it was much more likely to detect an obstructive disease requiring revascularization.

The forecast was the first randomized trial to assess the effectiveness of CCTA with targeted FFRCT for stable chest pain patients [97]. It was the second major study with the primary outcome of determining the role of FFRCT in reducing medical expenses compared to the standard strategy. However, the study also demonstrated that patients assessed with FFRCT had a 22% lower likelihood of undergoing ICA than the standard care group. Furthermore, those who received FFRCT evaluation had a 52% lower chance of undergoing ICA with a final result of no obstructive lesion. Therefore, FFRCT patients appear to have clinical outcomes and quality of life comparable to those who receive standard care, besides undergoing less invasive procedures.

The precise Trial is the latest randomized clinical trial that has evaluated the use of FFRCT in clinical practice [98]. This trial included participants from 65 North American and European centers who had stable symptoms of suspected CAD with no previous testing. These patients were randomly allocated to either the precision strategy (PS) or the usual testing (UT) group in a 1:1 ratio. The Prospective Multicenter Imaging Study for the Evaluation of Chest Pain (PROMISE) minimal risk score was used to objectively identify individuals with minimal risk for postponed testing. Those who did not fall into the minimal-risk category were assigned to have CCTA with selective FFRCT. UT included either stress testing or catheterization. Based on the study results, a greater number of the patients in the UT group had catheterization. However, only a few exhibited obstructive CAD, and revascularization was more often conducted in the PS group compared to the UT group. The precise Trial demonstrated that implementing an initial diagnostic strategy for stable chest pain, which involves assessing the risk level using quantitative methods and deferring testing for patients with minor risk, and using CCTA with selective FFR-CT for all other patients, resulted in improved clinical efficiency compared to the usual treatment approach at the one year.

### 5.2 Reducing the Medical Costs

In 2017, the United Kingdom Medical Technologies Guidance on FFRCT declared that implementing CCTA with FFRCT was projected to result in significant cost savings for the National Health Service [99]. Additionally, according to an economic analysis of the observational platform trial [100], FFRCT resulted in cost savings when an invasive procedure was planned but had no effect on costs when a non-invasive strategy was picked.

The forecast trial [97] aimed to assess whether utilizing FFRCT would lead to significant cost savings. However, the trial could not detect any difference in total expenses compared to the standard care plan. The low rate of scheduled initial ICA (7% of the study participants) and the extensive use of CCTA as the arranged initial test (65% of the study participants) may be responsible for this unfavorable result. Additionally, the fact that the costs in this trial were calculated according to UK National Health Service tariffs indicates the results may not be comparable to other countries with various cost structures in their healthcare systems, revealing another significant limitation of the FORECAST trial. As a result, further studies with larger populations in various countries with different healthcare tariffs may still be needed to investigate the role of FFRCT in reducing healthcare expenses.

## 6 LIMITATIONS

FFRCT has several benefits in clinical practice but has also faced criticism due to its limitations. FFRCT is impacted by CT-imaging challenges like poor image quality, motion artifacts, and image noise.

Since FFRCT depends on high-quality imaging, a substantial proportion of patient data sets have been discovered inappropriate for FFRCT assessment in clinical studies. In the NXT [17] and platform [33] trials, about 12% of subjects were excluded from the analysis. Also, 33% of CCTA datasets were excluded from the promise trial sub-study [101]. However, the latter was a retrospective study, and FFRCT calculation was not a goal of the initial investigation. In the advance trial and a major clinical cohort, 2.9% and 8.4% of CCTA data were rejected for FFRCT analysis, respectively [102]. Due to the increased usage of dual-source technology and enhanced algorithms, the rejection rates of these two later studies are lower than the others. Of note, in around 80% of the cases that had been disqualified, motion artifact was the primary cause of inappropriate imaging quality [102].

Multivariate analysis showed that several factors, including smoking, Tachycardia, a detector coverage of less than 16 cm, temporal resolution of more than 100 ms, and tube voltage of more than 120 kVp, were independently associated with increasing rejection rate [102].

Thus, strategies like administering medications during the procedure to lower heart rate (beta-blockers) and dilate coronary arteries (sublingual nitroglycerin), in addition to the use of more innovative scanners, were developed as an effective solution for the problems mentioned above [103, 104].

Calcification of the coronary arteries, on the other hand, may obscure the lumen and make the borders unclear for CFD evaluation. There was a trend toward reducing diagnostic accuracy with increasing Agatston score in the NXT sub-analysis, but the change was not statistically significant [105]. However, this can be attributed to the low percentage of patients with extremely high Agatston scores in this study. The accuracy of the FFRCT findings is further compromised by the variable microcirculation responses to the vasodilator drugs and physiological factors that affect fluid density and viscosity in patients.

Although FFRCT is helpful in determining the functional significance of coronary artery lesions, Myocardial ischemia burden still must be assessed by functional imaging, such as SPECT, PET-CT, CMR, or stress echocardiography since FFRCT does not provide this information. Additionally, FFRCT does not offer viability data that could help determine which patients need to be candidates for PCI. So, without this essential information, deciding whether a patient is a good candidate for revascularization only based on FFRCT results before conducting a secondary functional assessment might be challenging. The high price of HeartFlow, which costs around 1100 USD for each patient, is another issue. HeartFlow has limited application in acute situations due to its long computation time, ranging from 1 to 5 hours. While on-site options might save time, they have not yet been validated or made widely accessible. As a result, these concerns must be addressed in future studies before FFRCT may be used on a broad scale.

## CONCLUSION

FFRCT is a non-invasive tool that effectively evaluates the functional impact of stenosis in coronary arteries. It offers a dependable fractional flow reserve assessment by integrating anatomical data from CT scans with computational fluid dynamics. This allows healthcare providers to accurately detect lesions that restrict blood flow and make informed decisions about revascularization. FFRCT can reduce unnecessary invasive medical procedures and improve patient outcomes in various clinical scenarios. However, further research and clinical validation are necessary to enhance the role of FFRCT in current medical practice, leading to improved patient outcomes and better management of cardiovascular disease.

## AUTHORS' CONTRIBUTIONS

The authors confirm contribution to the paper as follows: study conception and design: Seyed Kianoosh Hosseini, drafting the manuscript: Roozbeh Narimani Javid; Designing figures and tables: Roozbeh Narimani Javid; Revising manuscript: Seyed Kianoosh Hosseini and Roozbeh Narimani Javid.

## Figures and Tables

**Table 1:** Summary of several studies utilizing different approaches to calculate the FFRCT.

**FFRCT Methods**					
**Study**	**Year**	**Calculation Algorithm**	**Computational Time**	**Diagnostic Accuracy**	**Population**
Nørgaard.*et al.* [17]	2014	HeartFlow 3D version of Navier-Stokes based on CFD	1-4 hour	Sensitivity 86% Specificity 79%	254 patients scheduled to undergo clinically indicated ICA for suspected CAD
Kruk. *et al.* [10]	2016	Siemens reduced-order(1D) version of navier-Stokes based on CFD	23.9±11.2 min	Sensitivity 75.6% Specificity 71.4%	96 lesions in 90 patients who underwent CTA for suspected CAD and were diagnosed with at least 1 intermediate coronary stenosis
Ko. *et al.* [106]	2017	Toshiba reduced-order(1D) version of navier-stokes based on CFD	27±7.5 min	Sensitivity 77.8% Specificity 86.8%	42 Symptomatic patients with no known CAD who were at intermediate or high risk and were scheduled for clinically mandated elective ICA
Itu. *et al.* [14]	2016	Siemens machine-learning software	2.4 sec	Sensitivity 81.6% Specificity 83.9%	87 patients and 125 lesions
Kamamaru. *et al.* [107]	2020	3D deep-learning-based algorithm	In seconds	Sensitivity 85.8% Specificity 63.4%	1052 patients included 131 patients whose CCTA studies showed 30-90% stenosis and underwent invasive FFR, and 921 patients who underwent clinically indicated CCTA without invasive FFR

## LIST OF ABBREVIATIONS

NPV: Negative Predictive Value
CCTA: Coronary Computed Tomography Angiography
CAD: Coronary Artery Disease
FFR: Fractional Flow Reserve
FFRCT: Fractional Flow Reserve Computed Tomography
DSE: Dobutamine Stress Echocardiography
